# Effect of duloxetine premedication for postherpetic neuralgia within 72 h of herpes zoster reactivation [PROCESS]: a study protocol for a randomized controlled trial

**DOI:** 10.1186/s13063-020-04919-6

**Published:** 2020-12-09

**Authors:** Zheng Chen, Niti Shrestha, Chunmei Zhao, Bifa Fan, Fang Luo

**Affiliations:** 1 Department of Pain Management, Beijing Tiantan Hospital, Capital Medical University, Beijing, China; 2 National Pain Management and Research Center, China-Japan Friendship Hospital, Beijing, China

**Keywords:** Postherpetic neuralgia, Duloxetine, Herpes zoster, Prevention

## Abstract

**Background:**

Postherpetic neuralgia (PHN) is the most common complication attributed to herpes zoster, which involves the reactivation of residual varicella zoster virus. It has been reported previously that pre-emptive amitriptyline following acute herpes zoster has shown latent positive effects in the prevention of PHN. In this study, by interfering with the same targets, norepinephrine and serotonin, we aim to evaluate whether pre-emptive duloxetine may proactively prevent PHN development.

**Methods:**

This is a nationwide multicentric, randomized, open-label, blinded-endpoint study that will recruit 750 participants from 18 primary centres in China. Patients aged more than 50 years who are diagnosed with uncomplicated HZ, present with vesicles within 72 h of their emergence, and have an average pain score of at least 40/100 mm on a visual analogue scale (VAS, 0 mm = no pain, 100 mm = worst possible pain, at opposite ends of a 100-mm line) will be recruited for this study. Participants will be randomized into a duloxetine arm and a control arm. Participants allocated to the duloxetine arm will be given antivirals, analgesics and duloxetine, while those allocated to the control arm will receive antivirals and analgesics but no duloxetine. The primary outcome of this study is preventive efficacy against PHN, which will be evaluated based on a 100 mm VAS. Any pain scores other than 0 mm on the VAS 12 weeks after HZ onset will be defined as PHN. The secondary outcomes will consist of the average weekly VAS score, the average weekly consumption of each analgesic, weekly feature of the pain, patients’ quality of life based on the 12-item Short-Form Health Survey, Patient Global Impression of Change Scale, sleep quality as evaluated by the Pittsburgh Sleep Quality Index and adverse events during the study period.

**Discussion:**

This study will investigate a prophylactic approach for reducing the prevalence of postherpetic neuralgia with duloxetine and will add significant new knowledge on the preventive effects of duloxetine on PHN.

**Trial registration:**

Clinicaltrials.gov NCT04313335. Registered on 18 March 2020.

## Administrative information

Note: the numbers in curly brackets in this protocol refer to SPIRIT checklist item numbers. The order of the items has been modified to group similar items (see http://www.equator-network.org/reporting-guidelines/spirit-2013-statement-defining-standard-protocol-items-for-clinical-trials/).

**Table:** 

Title {1}	Effect of Duloxetine Premedication for Postherpetic Neuralgia within 72 hours of Herpes Zoster Reactivation [PROCESS]: A Study Protocol for a Randomized Controlled Trial
Trial registration {2a and 2b}.	Clinicaltrials.gov, NCT04313335. Registered on March 18, 2020 *https://clinicaltrials.gov/ct2/show/NCT04313335*
Protocol version {3}	2020/04/30 Protocol Version 1.2
Funding {4}	None
Author details {5a}	Zheng Chen*, Department of Pain Management, Beijing Tiantan Hospital, Capital Medical University, Beijing, China. Niti Shrestha*, Department of Pain Management, Beijing Tiantan Hospital, Capital Medical University, Beijing, China. Chunmei Zhao*, Department of Pain Management, Beijing Tiantan Hospital, Capital Medical University, Beijing, China. Bifa Fan, National Pain Management and Research Center, China-Japan Friendship Hospital, Beijing, China. Fang Luo, Department of Pain Management, Beijing Tiantan Hospital, Capital Medical University, Beijing, China.
Name and contact information for the trial sponsor {5b}	Not applicable as this study has no external funding.
Role of sponsor {5c}	Not applicable as this study has no external funding.

## Introduction

### Background and rationale {6a}

Postherpetic neuralgia (PHN) is the most common complication attributed to herpes zoster (HZ), which involves the reactivation of residual varicella zoster virus (VZV). Although the definition varies, pain that persists for 4 weeks to 6 months following acute HZ onset is recognized as PHN. Risk factors [1] for the development of PHN include older age, skin damage and pain intensity during acute HZ. In China, the prevalence of HZ is reported to be 7.7% [2] among the adult population, and an estimated 2.77 [3] million cases are diagnosed annually. Among those cases, 30% [2] are predicted to develop PHN after 12 weeks. Recent investigation [4] has shown that PHN is associated with sleep disturbances, lower quality of life, restricted daily activities and increased medical costs, all of which impose heavy burden on the patient and their finances. In addition, neuropathic pain [5] often persists for months or years and is refractory to drugs. Although a group of medications such as antidepressants, antiepileptics and topical agents are recommended by international guidelines, management of PHN is still challenging. Current therapeutic modalities fail to meet clinical expectations, as significant pain relief is achieved in less than 50% of patients [6] even with the most effective medications for PHN. Preventive and pre-emptive management [7] has been advocated for PHN in the future.

As to PHN prevention, HZ vaccination has been commonly administered [8] in the aged population. The efficacy of this treatment has been indicated and well-conducted [9–11]. When administered prior to VZV reactivation, the HZ vaccine significantly reduces the incidence of HZ and PHN in the following years. Treatment for acute HZ is believed [12] to reduce the incidence of PHN by interfering with virus duplication, the inflammatory response and peripheral or central nociceptor activity. Antivirals [13] are recommended for acute HZ in certain non-immunocompromised patients, which are believed to repress viral replication. Simultaneously, the process of lesion healing is accelerated, and new lesion formation is reduced. Based on a systematic review, pain during acute HZ onset is alleviated by the administration of antivirals, while PHN remains [14]. Corticosteroid therapy [13], although controversial, is an alternative adjuvant to antivirals in acute HZ. Studies [15, 16] have shown that corticosteroids are related to early healing, improved daily activity and reduced pain intensity. However, another systematic review has shown that corticosteroids have a limited ability to decrease the incidence of PHN. The voltage-gated calcium channel blocker gabapentin is recommended as the first-line medication [7] for PHN and has been extensively applied in clinical practice. In a recent retrospective study [17], premedication with gabapentin administered for other peripheral neuropathies, including neuropathy caused by diabetes, adverse drug effects, radiotherapy and other diseases, before acute HZ onset was found to assist in the prevention of PHN. A similar outcome was observed in an uncontrolled open-label study [18]. The incidence of PHN was decreased after combination treatment with gabapentin and valacyclovir in patients with acute HZ. In contrast, Bulilete et al. [19] found no evidence that 1 week of valacyclovir (1000 mg tid) plus 5 weeks of gabapentin (titrated from 300 to 1800 mg per day) beginning within 3 days after HZ onset helped prevent PHN. Lee et al. [20] found that 12 weeks of gabapentin (900 mg per day) beginning within 4 days after HZ onset failed to reduce the incidence of PHN. Amitriptyline is a tricyclic antidepressant (TCA) that increases the availability of both norepinephrine and serotonin. A previous randomized study [21], in which amitriptyline administration was combined with antivirals for 90 days after acute HZ diagnosis, showed that the combined treatment did not achieve a significant preventive effect on PHN at 3 months after HZ onset compared to placebo. However, a delayed reduction in PHN incidence was found for amitriptyline at 6 months after HZ onset. In summary, beyond the vaccine, there is little evidence of a method to reduce the incidence of PHN after VZV reactivation. Therefore, convincing preventive approaches that begin after VZV reactivation are needed for researchers and clinicians.

Existing evidence [22] suggests that the transformation of HZ to PHN involves neural sensitization. Abnormal functional and structural changes have been observed in PHN brains and reported in recent studies [22]. These changes are believed to be closely related to the chronification of PHN. Descending inhibitory serotonergic and noradrenergic pathways [23] regulate the neural excitability of the dorsal horn. The inhibitory balance shifts in the context of neuropathic pain. Duloxetine has been approved for the treatment of neuropathic pain and recommended as the first-line [24] pharmacotherapy. The mechanism of action of duloxetine regarding the inhibition of serotonin and norepinephrine reuptake may enforce or reinforce the inhibitory mechanism, maintain or reconstruct inhibitory balance and potentiate pain alleviation or the avoidance of persistent pain induction.

In a previous study, pre-emptive amitriptyline has shown latent preventive effects on PHN by interfering with central reuptake of norepinephrine and serotonin, although there was no statistical difference in the reduction of incidence of PHN until 6 months after VZV reactivation. The limited sample size and unstandardized antiviral therapy seemed to have had an impact on the preventive efficacy. Consequently, we speculate that pre-emptive duloxetine, which has similar mechanisms and is safer in older patients [25], provided shortly after VZV reactivation may proactively stabilize the inhibitory balance before sensitization occurs. If successful, this stabilization will help reduce the incidence of PHN. We hypothesized that positive outcomes will be concluded upon enlarged sample size and standardized antiviral therapy. Thus, this multicentric, randomized, open-label, blinded-endpoint study was designed to evaluate whether premedication with duloxetine within 72 h after VZV reactivation has a promising preventive effect on PHN.

### Objectives {7}

The aim of this study is to evaluate whether premedication with duloxetine within 72 h after VZV reactivation has a promising preventive effect on PHN.

### Trial design {8}

**Fig. 1:**
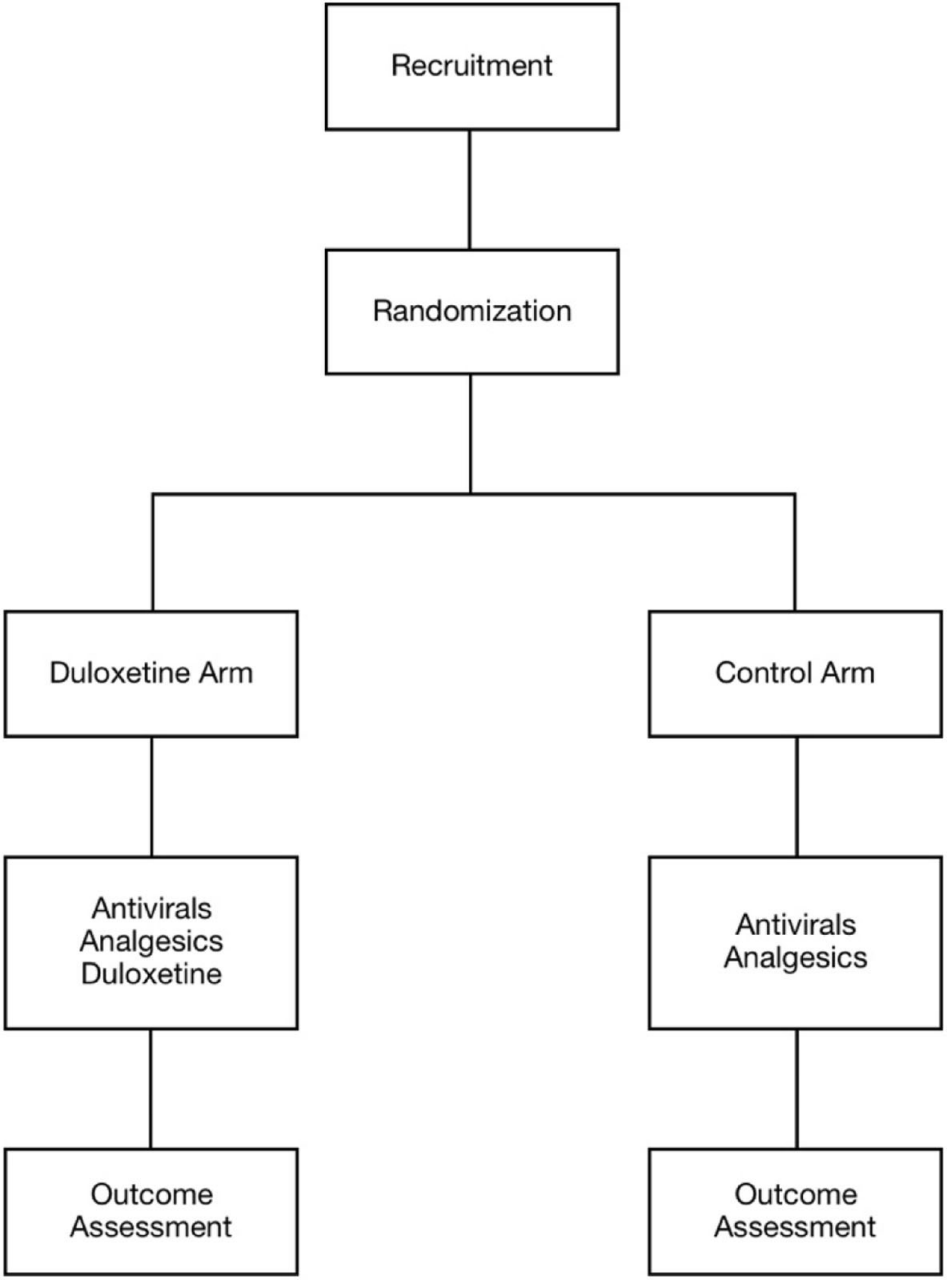
The timeline and detailed schedule of the PROCESS trial

This PROCESS trial is a multicentric, randomized, open-label, blinded-endpoint study, designed to evaluate whether premedication with duloxetine within 72 h after VZV reactivation has a promising preventive effect on PHN. In total, 750 participants will be recruited from 18 different hospitals all over China. Participants will be assigned to either the duloxetine arm or the control arm in an allocation ratio of 1:1. The timeline and detailed schedule of the study are illustrated and summarized in Fig. 1.

## Methods: participants, interventions and outcomes

### Study setting {9}

We designed a nationwide multicentric, randomized, open-label, blinded- endpoint study that will recruit at least 750 participants from Beijing Tiantan Hospital, China-Japan Friendship Hospital, The Second Hospital of Tianjin Medical University, Taiyuan Central Hospital of Shanxi Medical University, Linfen Fourth People’s Hospital, Shandong Provincial Hospital, Qingdao Municipal Hospital (Group), Huai’an Second People’s Hospital, The First Hospital of China Medical University, The Second Affiliated Hospital of Zhengzhou University, Fujian Provincial Hospital, Cangzhou Central Hospital, The People’s Hospital of Fujian Province, Beijing Tsinghua Chang Gung Hospital, Peking University International Hospital, Tianjin First Central Hospital, Tianjin Huanhu Hospital and Baoding First Central Hospital in 10 provinces and cities in China. We plan to begin trial recruitment on August 1, 2020. We estimate that the study will need to continue for 3 years to reach the intended goal of 750 participants. A list of study centres can be obtained from the clinical trials registry website: https://clinicaltrials.gov/ct2/show/NCT04313335

### Eligibility criteria {10}

Patients presenting to the Department of Emergency, Dermatology, Neurology or Pain Management at any participating centre will be screened for eligibility to join the present study. Patients aged more than 50 years who are diagnosed with uncomplicated HZ, present with vesicles within 72 h of their emergence, and have an average pain score of at least 40/100 mm on a visual analogue scale (VAS, 0 = no pain, 100 = worst possible pain, at opposite ends of a 100-mm line) will be considered to fulfil the inclusion criteria. Patients who refuse to participate in the study or to provide written informed consent; those who have a raw score of more than 50 points on the Zung Self-Rating Depression Scale [26]; those who are confirmed to have HZ with head, neck, ocular, mucous membrane, cranial nerve, or central nervous system involvement or generalized HZ; those who are observed to have haemorrhagic or necrotizing lesions, satellite lesions, abnormal vesicles or acute retinal necrosis; those who have been on immunosuppressive therapy or mono- or multi-pharmacotherapy that involves any tricyclic antidepressant, valacyclovir, duloxetine or cytotoxic medications before acute HZ onset; those who have been diagnosed with hepatic, renal or immune dysfunction; those who are pregnant or breastfeeding; those with recorded hypersensitivity to the study drugs; those with contraindications to valacyclovir or duloxetine; and those who have been immunized with the HZ vaccination will be excluded from the study. Prior to the initiation of this study, research assistants (at least 2 research assistants per centre) will be trained regarding the details of this study protocol to ensure that the work would proceed smoothly. When patients are identified and diagnosed with acute HZ, research assistants will be informed and asked to screen the patients for eligibility. Eligible patients who are willing to participate will be recruited for the study.

### Who will take informed consent? {26a}

Prior to the initiation of the study, research assistants (at least 2 research assistants per centre) will be trained regarding the details of this study protocol, to ensure smooth operations and consistency. Protocol modifications are not to be expected. When patients are identified and diagnosed with acute HZ, research assistants will be informed and asked to screen patients for eligibility. Eligible patients who are willing to participate will be provided a verbal explanation of the written consent. Patient queries will be answered and each patient interested in the study will have sufficient time to decide whether to participate in this study, and written informed consent will be obtained from willing participants.

### Additional consent provisions for collection and use of participant data and biological specimens {26b}

The consent form will include the permission of collection and use of participant data, should the participants decide to withdraw from the trial, along with the permission to share relevant data with members of regulatory authorities, when necessary. This trial does not involve the collection of biological specimens.

## Interventions

### Explanation for the choice of comparators {6b}

For patients assigned to the control arm, 1000 mg valacyclovir will be administered three times a day for 7 days. The antiviral therapy has been recommended as a standard therapy for HZ and widely applied in previous studies [18, 19, 21]. The mechanism involves hastening the resolution of lesions, preventing the formation of new lesions and decreasing the severity of acute pain [13]. The analgesics, including non-opiates, weak opiates and strong opiates, will be provided as needed.

### Intervention description {11a}

For participants assigned to the duloxetine arm, 20 mg duloxetine (YouBiLuo®, NHWA, China) will be orally administered once daily, after breakfast. If the patient’s VAS score reaches 0 within the first week, duloxetine will be discontinued; otherwise, it will be increased to 40 mg once daily, for the second week. If the VAS score reaches 0 within the second week, duloxetine will be tapered down to 20 mg once daily for a week and discontinued; otherwise, it will be increased to 60 mg once daily and maintained until a VAS score of 0 is obtained, or until the end of the 10th week. Then, duloxetine will be tapered to 40 mg once daily for a week, followed by 20 mg once daily for a week, and then discontinued. For participants who develop adverse effects, the dose will be adjusted to the last tolerated level. Participants in the control arm will not be given duloxetine during the 12-week treatment period.

### Criteria for discontinuing or modifying allocated interventions {11b}

All the AEs (adverse events) of the study will be closely monitored, properly documented in detail, and reported to the ethics committee with the aims of resolving the condition or even dissolution of the study if necessary.

### Strategies to improve adherence to interventions {11c}

Once assigned to one of the two study groups, a pharmacological protocol will be given as per allocation. Physicians and research assistants will be responsible for instructing participants to follow the protocols correctly, and to ensure adherence to interventions. After recruitment, each participant will be given a pain diary (PD), in which all outcomes can be recorded by the participants themselves during the study period. The specific recording criteria are as follows: (i) the VAS score will be recorded every morning before breakfast, (ii) AEs and consumption of each analgesic will be recorded every evening before bed, and (iii) the 12-item Short-Form Health Survey (SF-12), Patient Global Impression of Change Scale (PGIC) and Sleep quality as measured by the Pittsburgh Sleep Quality Index (PSQI) will be self-assessed at baseline and upon completion of weeks 4, 8, and 12. During the study period, the research assistants will monitor the participants’ completion of the PDs and deliver reminders as necessary through telephone calls or social media. At the end of week 12, the PDs will be collected by the research assistants and stored by the Data Safety Monitoring Board (DSMB). The research assistants will be available 24 h a day, 7 days a week, to answer any questions related to the study.

### Relevant concomitant care permitted or prohibited during the trial {11d}

For all participants, 1000 mg valacyclovir will be administered three times a day for 7 days, and analgesics will be given according to the three-step World Health Organization (WHO) pain ladder [27]. Non-opioids will be preferred for mild pain, weak opioids plus non-opioid analgesics will be recommended if moderate pain is developed, and strong opioids plus non-opioids will be provided for severe pain. All selected analgesics will be administered until *VAS* ***=*** *0*. Once VAS = 0, analgesics will be discontinued. Treatments not mentioned in this protocol will not be allowed unless approved and standardized by the research team. All unmentioned treatments will be recorded thoroughly by the research assistants.

### Provisions for post-trial care {30}

Hyponatremia, delirium and haemodynamic instability during the study period are anticipated for the participants of this trial.

### Outcomes {12}

#### Primary outcomes

The primary outcome is the preventive efficacy of PHN, which will be measured based on a 100 mm VAS in which 0 mm represents no pain and 100 mm represents the most severe pain imaginable. Any pain scores other than 0 mm on the VAS 12 weeks after HZ onset will be defined as PHN.

#### Secondary outcomes

1. Average weekly VAS score
2. Average weekly consumption per analgesics
3. Weekly feature of the pain
4. Quality of life (QoL) based on the 12-item Short-Form Health Survey (SF-12) [28] upon completion of weeks 4, 8 and 12
5. Patient Global Impression of Change Scale (PGIC) [29] at week 12
6. Sleep quality as measured by the Pittsburgh Sleep Quality Index (PSQI) [30] self-rated questionnaire upon completion of weeks 4, 8 and 12

### Participant timeline {13}

Participant timeline is shown in Table 1.

**Table 1:**
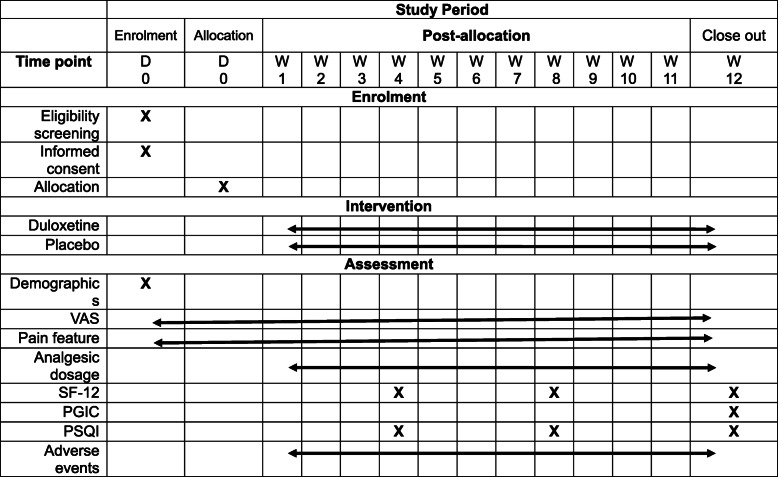
Time schedule of participant enrolment, allocation and assessment

*VAS* visual analogue scale, *QoL* quality of life, *SF-12* 12-item Short-Form Health Survey, *PGIC* Patient Global Impression of Change Scale, P*SQI* Pittsburgh Sleep Quality Index, *D* day, *W* week

### Sample size {14}

Based on the reported prevalence, the incidence of PHN in China is approximately 30% [2] after VZV reactivation. We predict that the same incidence will be observed in participants from the control arm. For participants in the duloxetine arm, the preventive protocol is expected to reduce the incidence to 20%. Assuming a 15% rate of loss to follow-up, a total of 750 participants would provide 90% power to detect the significant difference in the incidence of PHN using a 2-tailed alpha of 0.05.

### Recruitment {15}

This is a nationwide multicentric study that will recruit at least 750 participants from Beijing Tiantan Hospital, China-Japan Friendship Hospital, The Second Hospital of Tianjin Medical University, Taiyuan Central Hospital of Shanxi Medical University, Linfen Fourth People’s Hospital, Shandong Provincial Hospital, Qingdao Municipal Hospital (Group), Huai’an Second People’s Hospital, The First Hospital of China Medical University, The Second Affiliated Hospital of Zhengzhou University, Fujian Provincial Hospital, Cangzhou Central Hospital, The People’s Hospital of Fujian Province, Beijing Tsinghua Chang Gung Hospital, Peking University International Hospital, Tianjin First Central Hospital, Tianjin Huanhu Hospital and Baoding First Central Hospital in 10 provinces and cities in China. Patients presenting to the Department of Emergency, Dermatology, Neurology or Pain Management at the aforementioned participating centres will be screened for eligibility.

Prior to the initiation of the study, research assistants (at least 2 research assistants per centre) will be trained regarding the details of this study protocol, to ensure smooth operations and consistency. Patients aged more than 50 years who are diagnosed with uncomplicated HZ, who present with vesicles within 72 h of their emergence, and have an average pain score of at least 40/100 mm on a visual analogue scale will be considered for enrollment. The research assistants will be informed and asked to screen patients for eligibility. Eligible patients who are willing to participate will be provided with a verbal explanation of the written consent. Patient queries will be answered and each patient interested in the study will have sufficient time to decide whether to participate in this study, and informed consent will be obtained from willing participants.

### Assignment of interventions: allocation

#### Sequence generation {16a}

After signing the written informed consent, participants will be randomized for allocation. Randomization will be performed using a web-based central randomization system with permuted blocks by an independent research assistant. Participants will be assigned to either the duloxetine arm or the control arm in an allocation ratio of 1:1. Given the open-label nature of this study, the patients, physicians and research team will inevitably be aware of the allocation. However, the outcome assessors will be blinded to the intervention.

#### Concealment mechanism {16b}

Since this is an open-label study, the patients, physicians and research team will inevitably be aware of the allocation. However, the outcome assessors and statistical analysts will be blinded to the intervention.

#### Implementation {16c}

An experienced research investigator, not involved in any other aspect of the study will use a web-based central randomization system with permuted blocks, to assign the patients in either the duloxetine arm or the control arm in a 1:1 ratio. Participants who fulfil the eligibility criteria will be enrolled in the study by the research assistants assigned for recruitment.

### Assignment of interventions: blinding

#### Who will be blinded {17a}

Only the outcome assessors and data analysts will be blinded to the study.

#### Procedure for unblinding if needed {17b}

This is an open-label study with only the outcome assessors and data analysts being blinded. Therefore, unblinding will not occur.

### Data collection and management

#### Plans for assessment and collection of outcomes {18a}

Research assistants will gather the patients’ baseline demographic characteristics searching the institutional electronic medical records or asking participants directly. The baseline and outcomes for the intervention will be compared to the control arm for primary analysis. After recruitment, each participant will be given a PD, in which all outcomes can be recorded by the participants themselves during the study period. The specific recording criteria are as follows: (i) the VAS score will be recorded every morning before breakfast, (ii) AEs and consumption of each analgesic will be recorded every evening before bed, and (iii) the SF-12, PGIC and PSQI will be self-administered at baseline and upon completion of weeks 4, 8 and 12. During the study period, the research assistants will monitor the participants’ completion of the PDs and deliver reminders as necessary through telephone calls or social media software. At the end of week 12, the PDs will be collected by the research assistants and stored by the DSMB. An investigator will sample every participant file and check for missing data.

#### Plans to promote participant retention and complete follow-up {18b}

Each participant will be given a PD, in which all outcomes can be recorded by the participants themselves throughout the study period. The research assistants will be available 24 h a day, 7 days a week, to answer any questions related to the study. During the study period of 12 weeks, the research assistants will monitor the participants’ completion of the PDs and deliver reminders as necessary through telephone calls or social media software.

#### Data management {19}

The institutional data monitoring committee will serve as the DSMB and be responsible for data safety. The study protocol will be sent to the DSMB before the initiation of the study. During the study period of 12 weeks, the research assistants will monitor the participants’ completion of the PDs. At the completion of 12 weeks, a data quality audit will be performed. All PDs collected will be stored in a secure location by the lead investigators, undisclosed to other research members.

#### Confidentiality {27}

Throughout the duration of the study, all patient data will be recorded and stored in a locked cabinet by the lead investigator. Patient information will be kept confidential and only the lead investigator of each assigned centre will have access to the files corresponding to the personal data of the respective participants.

#### Plans for collection, laboratory evaluation and storage of biological specimens for genetic or molecular analysis in this trial/future use {33}

This study does not involve collecting, laboratory evaluation or storage of biological specimens for genetic or molecular analysis for future use.

### Statistical methods

#### Statistical methods for primary and secondary outcomes {20a}

Statistical analyses will be performed using the SPSS 25.0 software package for Windows (SPSS, Inc., Chicago, IL, USA). The baseline and outcomes for the intervention will be compared to the control arm for primary analysis. Continuous variables will be expressed as the mean (SD) or median (IQR) and compared using Student’s *t* test or the Mann-Whitney *U* test. Normality will be tested using the Kolmogorov-Smirnov test. Categorical variables will be expressed as the number of patients (percentage) and compared using the chi-squared test or Fisher’s exact test. A 2-tailed alpha of 0.05 will be used as the threshold of statistical significance. The incidence of PHN in each arm at the end of week 12 will be compared using the chi-squared test. Subgroup analyses will be performed for age, gender, time to medication, maximum duloxetine dosage and average weekly pain score. Multiple logistic regression will be used to evaluate and determine the interference of the above confounding factors in the primary outcome. All analyses will be performed using both the intention-to-treat and per-protocol principles, and the final conclusion will be determined in line with the intention-to-treat principle.

#### Interim analyses {21b}

Any AE or SAE (serious adverse events) will be reported to the DSMB and the IRB (Institutional Review Board) for judgement. Anticipated severe problems that may be detrimental to the participants include rapid-onset hyponatremia, delirium, haemodynamic instability. The aforementioned adverse events and other serious life-threatening adverse events leading to prolonged hospital stay or death will be reported to the IRB of the respective participating centre, and our study will be terminated immediately.

#### Methods for additional analyses (e.g. subgroup analyses) {20b}

Subgroup analyses will be performed for age, gender, time of medication, maximum duloxetine dosage and average weekly pain score. Multiple logistic regression will be used to evaluate and determine the interference of the above confounding factors in the primary outcome. All analyses will be performed using both the intention-to-treat and per-protocol principles, and the final conclusion will be in line with the intention-to-treat principle.

#### Methods in analysis to handle protocol non-adherence and any statistical methods to handle missing data {20c}

At least two research assistants from each centre will be trained in detail regarding the same protocol, before the implementation of this study. The research assistants will be readily available to answer any questions throughout the entire study period of 12 weeks, and monitor the participants’ completion of the PDs and deliver reminders as necessary through telephone calls or social media. PD recordings are mandatory and missing data are not to be expected. All outcomes will be analysed according to the intention-to-treat principle, and once enrolled, all patient data will be analysed irrespective of the findings.

#### Plans to give access to the full protocol, participant-level data and statistical code {31c}

Patient information will be kept confidential and only the lead investigator of each centre will have access to the files corresponding to the personal data of the respective participants. After completion of the study, the results will be made public through publication in a scientific journal along with conferences related to pain management, and the clinicaltrials.gov website. Necessary data will also be considered to be made available from the corresponding author on reasonable request.

### Oversight and monitoring

#### Composition of the coordinating centre and trial steering committee {5d}

The coordinating centre (CC) will be responsible for the training of all research assistants and the coordination of all standardized quality control aspects such as PDs, operation manual and forms. It will include a Principal Investigator (PI), two expert pain management specialists, a pain management nurse and a statistician. It will serve as a link between all the 18 centres involved in the study, the research assistants involved in recruitment, assistance and follow-up.

The trial steering committee (TSC) will include the PI, an independent chair, two independent pain physicians and an independent statistician. It will supervise the trial progress, resolve unanticipated complications if any and assess AE and SAE and their potential correlation with the study drugs. It will supervise the work of various subcommittees such as quality control subcommittee for the training of research assistants, subcommittee of research assistants involved in recruitment and clinical activities, subcommittee for publications and presentations at the completion of the study. The TSC will report any unforeseen events and report to the IRB. The IRB of Beijing Tiantan Hospital will be supervising the trial and meet at least annually to oversee the study conduct and progress.

#### Composition of the data monitoring committee, its role and reporting structure {21a}

The data monitoring committee (DMC) comprised of two statisticians and a pain physician will perform independent review of data outcomes and patient safety. It will meet biannually and report to the TSC.

#### Adverse event reporting and harms {22}

The TSC will conduct regular inspections of the trial progress and report any unforeseen events to the IRB. Any AE or SAE (serious adverse events) will be reported to the IRB (Institutional Review Board) for judgement. Anticipated severe problems that may be detrimental to the participants involve rapid-onset hyponatremia, delirium and haemodynamic instability. Such serious life-threatening adverse events as well as other severe complications leading to prolonged hospital stay or death will be reported to the IRB and our study will be terminated immediately.

#### Frequency and plans for auditing trial conduct {23}

The IRB will be making regular inspections of trial conduct. The progress report of this PROCESS trial will be submitted to the IRB 1 month ahead of the date, 2020/12/31, and will be reviewed annually.

#### Plans for communicating important protocol amendments to relevant parties (e.g. trial participants, ethical committees) {25}

Any deviations from the protocol will be fully documented in a breach report form, recorded in a protocol deviation log and reported to all relevant parties. Although protocol modifications are not to be expected, any modifications due to unforeseen circumstances will be reported to the regulatory bodies by sending updated versions of the protocol. A copy of the revised version will be stored by the PI and updated in the clinical trials registry website.

#### Dissemination plans {31a}

After completion of the study, the results will be made public through publication in a scientific journal along with conferences related to pain management, and the clinicaltrials.gov website.

## Discussion

This is a large randomized, multicentric, open-label, blinded-endpoint study aiming to test whether premedication with duloxetine within 72 h after VZV reactivation has a preventive effect against PHN. To the best of our knowledge, this study is the first to examine the preventive effects of duloxetine on PHN. PHN is the most common complication following acute HZ. Based on recent epidemiological studies, 61.8% to more than 70% of PHN cases in China develop in patients aged more than 50 years [3, 4]. The results of these studies were consistent with previous findings in western countries [6]. Patients older than 50 years have long been the main subjects for studies on PHN in prior clinical trials [19, 21]. For a pragmatic study design, only patients aged more than 50 years will be included in the present study. Duloxetine, a serotonin–norepinephrine reuptake inhibitor, was first approved for primary depression. In recent years, it has also been recommended as the first-line [24] therapy for neuropathic pain. The recommended usage of oral duloxetine is to start at 30 mg once a day and gradually increase by 30 mg each time until the optimal dosage. An alternative usage is to begin at 20 mg and increase by 20 mg each time until optimal dosage. In this study, the latter is chosen for its superior accuracy. The maximum dosage of duloxetine allowed is 120 mg per day. In the treatment of diabetic neuropathy and fibromyalgia, a higher dosage of duloxetine demonstrated no benefit compared to 60 mg per day and was not tolerated as well as the lower dose. On the other hand, pharmacodynamically, duloxetine inhibits the uptake of both serotonin and norepinephrine, of which the former is inhibited much more strongly than the latter. One study suggests that the inhibition of norepinephrine (NE) [31] is relatively weak if the daily oral dose of duloxetine is less than 60 mg. In our study, patients aged more than 50 years will be recruited. Hence, considering the safety issues and pharmacodynamic effectiveness, 60 mg per day will be set as the maximum dosage. Long-term tolerance has been proven by a previous study, in which duloxetine was administered at 60 mg twice per day for 52 [32] consecutive weeks. No severe drug-related side effects or AEs were observed. Notably, duloxetine was found with rapid-onset hyponatremia and delirium, according to a recent investigation [33]. Concerning events will be carefully monitored throughout the study period. Regarding drug interactions, duloxetine is not documented to be a contraindication to valacyclovir or vice versa. Similarly, based on our pilot study, no specific adverse events were observed for duloxetine in combination with valacyclovir, and no drug interactions were detected. During the study period, the principal investigators will monitor all AEs and SAEs in real time. The safety precautions in our study will be further adjusted based on the study process and AE reports.

This study has several limitations. An open-label design instead of a blinded, placebo-controlled design will be used in this nationwide multicentric study due to limited funding support. Although an open-label trial [34] reflects real clinical circumstances more effectively than a blinded trial and maintains the advantages of randomized allocation and blinded outcome assessors, the therapy recommended for the control arm can be considered an active control, as the intervention of duloxetine involves an add-on therapy, the effect of placebo may not be well perceived. As a result, VAS in the duloxetine arm may be underestimated; however, limited impact on primary outcome is assumed as any pain score other than 0 on the VAS at 12 weeks after HZ onset will be established as PHN. Although lower VAS is expected for the duloxetine arm, the incidence of PHN may not be significantly influenced by the absence of placebo. Even so, randomized, blinded, placebo-controlled clinical trials are needed. The study period in this trial is limited to 12 weeks; consequently, little is revealed about the long-term preventive effectiveness of duloxetine. Similarly, the maximum dosage of duloxetine was set at 60 mg per day, and this study does not address whether a higher dosage would have a more significant advantage or what dosage is optimal in the prevention of PHN. For safety reasons, immunosuppressed patients will not be included in the current study. Further investigation is needed for that population.

## Trial status

This protocol version 1.1 (2020/04/10) has been approve by the IRB of Beijing Tiantan Hospital Affiliated to Capital Medical University. We plan to begin trial recruitment on August 1, 2020. We estimate that the study will need to continue for 3 years to reach the intended goal of 750 participants. Hence, recruitment for this PROCESS trial is expected to complete by September 30, 2023.

## Abbreviations

PHN: Postherpetic neuralgia
HZ: Herpes zoster
VZV: Varicella zoster virus
TCA: Tricyclic antidepressants
VAS: Visual analogue scale
PD: Pain diary
AE: Adverse events
SAE: Serious adverse events
SF-12: Short-Form Health Survey
PGIC: Patient Global Impression of Change Scale
PSQI: Pittsburgh Sleep Quality Index
DSMB: Data Safety Monitoring Board
WHO: World Health Organization
QoL: Quality of life
IRB: Institutional Review Board
CC: Coordinating centre
PI: Principal Investigator
TSC: Trial steering committee
DMC: Data monitoring committee
NE: Norepinephrine
